# Numerical and Experimental Analysis of the Mode I Interlaminar Fracture Toughness in Multidirectional 3D-Printed Thermoplastic Composites Reinforced with Continuous Carbon Fiber

**DOI:** 10.3390/polym15102403

**Published:** 2023-05-22

**Authors:** Jonnathan D. Santos, José M. Guerrero, Norbert Blanco, Jorge I. Fajardo, César A. Paltán

**Affiliations:** 1 Grupo de Investigación en Nuevos Materiales y Procesos de Transformación (GIMAT), Universidad Politécnica Salesiana, Calle Vieja 12-30 y Elia Liut, Cuenca 010105, Ecuador; jfajardo@ups.edu.ec (J.I.F.); cpaltan@ups.edu.ec (C.A.P.); 2 Analysis and Advanced Materials for Structural Design (AMADE), Department of Mechanical Engineering and Industrial Construction, Universitat de Girona, Avda. M. Aurèlia Capmany 61, 17003 Girona, Spain; josemanuel.guerrero@udg.edu (J.M.G.); norbert.blanco@udg.edu (N.B.)

**Keywords:** 3D-printed composite, interlaminar fracture toughness, experimental characterization, finite element simulation, multidirectional interface

## Abstract

It is well known that the use of continuous reinforcing fibers can largely improve the typical low in-plane mechanical properties of 3D-printed parts. However, there is very limited research on the characterization of the interlaminar fracture toughness of 3D-printed composites. In this study, we investigated the feasibility of determining the mode I interlaminar fracture toughness of 3D-printed cFRP composites with multidirectional interfaces. First, elastic calculations and different FE simulations of Double Cantilever Beam (DCB) specimens (using cohesive elements for the delamination, in addition to an intralaminar ply failure criterion) were carried out to choose the best interface orientations and laminate configurations. The objective was to ensure a smooth and stable propagation of the interlaminar crack, while preventing asymmetrical delamination growth and plane migration, also known as crack jumping. Then, the best three specimen configurations were manufactured and tested experimentally to validate the simulation methodology. The experimental results confirmed that, with the appropriate stacking sequence for the specimen arms, it is possible to characterize the interlaminar fracture toughness in multidirectional 3D-printed composites under mode I. The experimental results also show that both initiation and propagation values of the mode I fracture toughness depend on the interface angles, although a clear tendency could not be established.

## 1. Introduction

High-performance fiber-reinforced composites offer high stiffness, a high strength to weight ratio, high thermal conductivity, good resistance to corrosion, etc. They are widely used in many of the different industrial sectors that play an important role in our economy, such as aeronautical and aerospace, automotive, sport, energy, construction, and defense industries. However, the traditional manufacturing techniques for fabricating composite materials and structural components require time-consuming tasks, such as manual layup for certain laminates, in addition to requiring expensive curing equipment and tooling, which are economically unsuitable for small batches of production. These limitations have promoted the further exploitation of Additive Manufacturing (AM), typically known as 3D printing. This technology is attractive for complementing the conventional methods for fabricating composites. In addition, it can be seen as a convenient alternative for manufacturing small quantities of a newly designed products, molds, or tooling, designed according to an optimal structure and mechanical performance while minimizing weight [1,2].

The first continuous Fiber-Reinforced Polymer (cFRP) composite printer was presented by Markforged^®^ (Watertown, MA, USA) in 2014 [3], and had a printing head with two separate extrusion nozzles; one for plastic, i.e., PolyAmide 6 (PA), and one for the reinforcing fiber supply (i.e., continuous carbon, glass, or Kevlar fibers), all of which were Markforged^®^ proprietary materials. This technology has received much attention from within both the industry and the scientific community because of its attractive advantages, such as high stiffness and strength, especially with respect to the Short Fiber-Reinforced Polymer (SFRP) composites, which can be manufactured with conventional 3D printers [1,4]. In fact, there is a relatively large number of published works on the experimental characterization of Markforged^®^ material. As Díaz-Rodríguez et al. [5] indicate, most of these studies have focused more on the tensile behavior [6,7,8,9,10,11] and less on bending [7,12,13,14,15], in-plane shear [16,17], interlaminar shear [18], impact [19] and fatigue and creep loading [20,21]. Nevertheless, there is still a lack of knowledge on the characterization of this material within several fields, such as the mechanical properties under degrading environments, out-of-plane shear, and fracture toughness [5,22]. Considering that the 3D printing of composites is based on the robotic deposition of adjacent filaments, the layer-by-layer ply might trigger a detrimental impact on the adhesion quality and the interlaminar fracture toughness. This is a relevant to the mechanical performance of 3D-printed cFRP composites, in that it can hinder a wider application. 

Although 3D-printing technology has been enhanced substantially in recent years, there is no specific standard for evaluating the fracture toughness of 3D-printed parts. Hence, the standards which are already available for plastic parts [22] (for example, compact tension (CT) [23,24,25], single-edge cracked [26,27], three-point bending [28,29], and Double Cantilever Beam (DCB) [30,31,32]) have been used for SFRP and cFRP composites as well. García-Guzman et al. [33] experimentally and analytically studied the fracture behavior of adhesively bonded DCB composite specimens made of Glass Fiber reinforced PolyAmide (GF/PA), with both trapezoidal and flat interface patterns. The samples were manufactured following the guidelines discussed by Cordisco et al. [34] using a Markforged MarkOne^®^ 3D printer. It was found that the fracture toughness for both onset and propagation was higher for the trapezoidal interface than for the conventional flat interface. The contribution of fracture in mode II along the trapezoidal path might explain the increasing experimental fracture results. The higher the ratio between amplitude and wave length (*A* = 1 mm amplitude and *λ* = 8 mm wavelength), the higher the fracture toughness (803% higher than the flat DCB). Iragi et al. [16] were the first to characterize the interlaminar fracture toughness in mode I and mode II for the Carbon Fiber reinforced PolyAmide (CF/PA) 3D printed composite. The DCB and End Notched Flexure (ENF) tests were performed in accordance with the guidelines of the corresponding ASTM standards [35,36]. To prevent the failure of the arms during the tests, 3 mm thick Dyneema^®^ doublers were bonded to the CF/PA specimens. It was found that, contrary to the common tendency in composite materials, the initiation fracture toughness in mode II (*G*_IIc_ = 1.59 kJ/m^2^) was lower than the fracture toughness in mode I (*G*_Ic_ = 2 kJ/m^2^). The authors ascribed this unusual material behavior to the large number of fiber bridges in mode I tests and to the lack of matrix shearing in mode II tests. Taking this into account, Santos et al. [17] characterized the interlaminar fracture toughness in mode I [37] of the same CF/PA composite, but considering two different specimen thicknesses, without sandwiching the beam arms with another composite or a doubler. They also considered two different tests to characterize the mode II fracture toughness [36,38] for both initiation and propagation. It was concluded that the use of thicker specimens is not appropriate for this material type, ascertaining that the initiation interlaminar fracture toughness of thinner specimen in mode I (*G*_Ic_ = 1.5 kJ/m^2^) was lower than the fracture toughness in mode II *(G*_IIc_ = 1.95 kJ/m^2^), which is the usual tendency for traditional composite materials. In the same vein, Polyzos et al. [39] characterized the initiation interlaminar fracture toughness in mode I [35] and mode II [38] of the same material, obtaining similar toughness values to Santos et al. [17], although thicker specimens were used for the DCB test. Goh et al. [40] explored the effects that the printing parameters have on the interlaminar fracture toughness of commercial cFRP composite. DCB samples were manufactured using the continuous CF/PA Markforged^®^ composite in an open-source 3D printer (Hello BeePrusa). It was found that by increasing the nozzle temperature, the fracture toughness of the material can increase by more than 200%. In fact, the highest mean fracture toughness was obtained with high nozzle and bed temperatures, and a low printing speed. On the other hand, the maximum mean value reported by Goh et al. (*G*_Ic_ = 943 J/m^2^) is lower than the values reported by other authors using the commercial Markforged MarkTwo^®^ printer [16,17,39]. Recently, the hybrid effect of cFRT composites was analyzed using a DCB test for CF/CF, CF/Kevlar, and Kevlar/Kevlar interfaces [41]. The propagation value of the interlaminar fracture toughness that was obtained revealed an improvement, ascribed to the hybridization. The toughness value obtained was 2705 J/m^2^ for the hybrid system, while for the CF/CF and Kevlar/Kevlar cases the value achieved was 889 and 3101 J/m^2^, respectively.

Khosravani et al. [42] used the Markforged MarkTwo^®^ printer to manufacture a Semi-Circular Bending (SCB) GF/PA specimen with a diameter of 60 mm, exploring the effect that volume fraction had on the stiffness and interlaminar strength. The authors concluded that the higher the fiber volume fraction, the higher the stiffness and the fracture toughness value. Wang et al. [43] studied the fracture response of both interlayer hybrid CF/PA and Kevlar Fiber reinforced PolyAmide (KF/PA) composites under a quasistatic indentation test, in accordance with the ASTM D6264 standard [44]. Two Kevlar and two carbon plies were located at three different thickness positions (top, middle, and bottom) using the Markforged Mark7^®^ printer. The reinforcement at the middle location showed the best response, delaying both the onset and the propagation of fracture across all configurations. The maximum indentation force was obtained when the Kevlar plies were stacked after the two carbon plies, both at the top and bottom locations. Huang and Joosten [45] investigated two mesoscale architectures (the cross-ply and the semiwoven), so as to characterize the CF/PA composites using the Compact Tension test. The initiation value of the translaminar fracture toughness of the semiwoven configuration (16.2 kJ/m^2^) was higher than that of the cross-ply one (14.4 kJ/m^2^). The opposite trend was observed for the propagation toughness values, which were 24.9 kJ/m^2^ and 37.5 kJ/m^2^, respectively. 

Although different aspects have been investigated in 3D-printed continuous-fiber-reinforced thermoplastic composites, to date, in-depth research about delamination in this type of material is still missing, especially for multidirectional fibrous systems. However, delamination is one of the most dangerous failure mechanisms for both 3D printed and traditionally laminated composites. Although fracture toughness is usually measured in a conservative way, using unidirectional continuous fiber systems, the orientation of the interface layers can significantly affect the toughness value [39,46]. In fact, this is a key material property needed for improving both the modeling and design of composite materials, both in general as well as in 3D printed parts. To the best of the authors’ knowledge, there is no study on the fracture onset and propagation of Multidirectional Double Cantilever Beam (MD-DCB) specimens that makes use of either numerical models or experimental campaigns. This study summarizes the work carried out by the authors to assess the feasibility of defining suitable stacking sequences for MD-DCB specimens, in addition to avoiding plane migration and asymmetric growth during the propagation of the interlaminar crack. First, elastic analysis with a finite element model (using cohesive elements for delamination and an intralaminar ply failure criterion) were carried out, to determine the most appropriate multidirectional interfaces. The analysis also investigated the effect the specimen stiffness had on the crack jumping and asymmetric crack propagation which occurred in MD-DCB tests. Second, after identifying the most attractive stacking sequences from the numerical simulation results, an experimental campaign was performed, to validate the methodology obtained in the numerical study. Bar one, all delamination tests avoided the crack jumping failure mode during opening, which validated the FE methodology. A fractographic analysis was carried out to analyze the morphology response of representative postmortem specimens. Similar optical characteristics were identified in all samples, although the orientation of the interface layers affected the failure mechanisms.

## 2. Design of Laminates

The continuous carbon-fiber-reinforced Polyamide 6 (CF/PA) composite from the Markforged MarkTwo^®^ was chosen to carry out the elastic and numerical analyses. This exploration, which considered several laminates, intended to study the effect that the flexural stiffness of the specimen arms had on both the geometry of the crack front and the onset of the matrix cracking ahead of the interlaminar crack tip as it propagated. Most mechanical properties were experimentally determined by the authors, in accordance with the appropriate ASTM and ISO standards [17]. Table 1 presents the material properties of the continuous carbon-fiber-reinforced thermoplastic composite.

### 2.1. Laminate Configurations and Elastic-Bending Properties 

For the definition of the stacking sequences of the specimens and their corresponding interface angles at the crack plane, it must be considered that a high mismatch angle can lead to a crack propagation that cannot be anticipated by the standard [37], which would thus invalidate the test. Taking this into account, the orientation for the plies at the interfaces has been limited to a combination of seven angles: 0, ±15, ±30, and ±45. Nevertheless, the repetition of angles at the two interfaces is excluded, as the real interest of the analysis is when two different orientations are present at the delamination interface.

In their numerical investigation, Sebaey et al. [47] concluded that for MD-DCB specimens of traditional composite laminates, the higher the flexural stiffness of the specimen arms, the higher the probability of obtaining a valid interlaminar fracture toughness characterization in mode I. However, in a previous study on unidirectional specimens with the same CF/PA composite as the one considered here [17], stable and smooth crack onset and propagation was only obtained for thin specimens with 1.5 mm thick arms. Specimens with 2.5 mm thick arms showed rapid crack propagation, stick–slip effect, and crack branching, which invalidated the test.

The initial laminate configuration for the MD-DCB specimens considered a total of 24 plies, 12 per specimen beam, for a total thickness of 3 mm. The stacking sequence of each specimen beam is symmetrical and balanced, with the inside plies oriented at 0°. Thus, the stacking sequence of the specimen corresponds to [(±θ_1_/0_4_)_s_//(±θ_2_/0_4_)_s_], where // indicates the location of the interface. For a better identification, the following codification was used: L1_12_12_θ_1__θ_2_, where L1 indicates the laminate series, the two ‘12’ numbers indicate the total number of plies in the bottom and top beams, respectively, and θ_1_ and θ_2_ denote the ply orientation of the layers adjacent to the interface, representing bottom and top, respectively.

During the propagation of an interlaminar crack in a DCB test, the uniformity of the energy release rate is favored using the minimization of two elastic parameters, *B*_t_ and *D*_c_. *D*_c_ indicates the curvature ascribable to longitudinal/transverse bending coupling, which should be less than 0.25 [48,49]. *B*_t_ indicates the skewness of the crack profile ascribable to bending/twisting coupling, which should be maintained as low as possible to ensure a symmetric crack front [50,51]. Hence, *B*_t_ and *D*_c_ depend on the bending stiffness matrix coefficients *D*_ij_, and can be determined as

(1) $$D_{c}=\frac{D_{12}^{2}}{D_{11}D_{22}}\;\mathrm{and}\;B_{t}=\frac{\left|D_{16}\right|}{D_{11}}$$

For this study, the laminate configuration of each DCB specimen was divided into three parts: the whole laminate (A), the lower arm (B), and the upper arm (T), as suggested by Prombut et al. [49]. The three parts should follow the same rules as the elastic, in order to achieve a symmetrical crack front. Moreover, we took into account that, in order to achieve mode I crack propagation, the opening of the two arms of the specimen should be symmetrical. Thus, the flexural stiffness of the two arms of the specimen should be as similar as possible. To verify this requirement, the flexural stiffness of the two arms of all the considered MD-DCB specimens were evaluated and compared, defining a flexural–stiffness ratio. This ratio corresponds to the ratio between the minimum flexural stiffness of the two arms divided by the maximum one. In this way, flexural–stiffness ratios close to one indicate similar flexural stiffness for both arms, and a high probability of symmetrical opening. Conversely, specimens with flexural–stiffness ratios lower than 0.9 should be avoided, as the probability of symmetrical opening is low.

The analysis of the *B*_t_ parameter for all the L1_12_12_θ_1__θ_2_ laminates, where θ_1_ and θ_2_ were varied according to the seven orientation angles previously described, determined to have a maximum value equal to 0.057 for the 45° plies. As expected, the lower the lamina orientation, the lower the value of *B*_t_. Considering that, while the value of this parameter should be as low as possible, no threshold value has been established, this elastic constant is not considered as decisive for assessing the DCB laminate configurations. Thus, only the bending/twisting parameter, *D*_c_, will be used to assess the appropriateness of the proposed DCB specimens. Table 2 summarizes the results of the evaluation of the *D*_c_ for the twelve pairs of interface angles for laminate L1.

As can be seen in Table 2, half of the interface configurations did not fulfill the condition, and the value of *D*_c_ was higher than the limit (0.25) in at least one beam of the specimen. Thus, a bending/twisting coupling could be expected during the test, and these laminate configurations were discarded. Thus, only L1 specimens with interfaces 0/15, 0/30, −15/15, −15/30, 15/30 and −30/30 were to be considered.

The analysis of the flexural stiffness of the 12 interfaces considered for laminate L1 (summarized in Appendix A for conciseness reasons), reveals that the minimum value of the flexural–stiffness ratio for the valid interfaces was 0.56. This ratio is only higher than 0.9 for interfaces −15/15 and −30/30, with a ratio of one, and interface 0/15, with a ratio of 0.9. Consequently, there were only three interface configurations for laminate type L1 with symmetrical opening and low bending/coupling effects. For this reason, more laminate configurations were taken into account, in order to increase the number of suitable interface configurations. Following the suggestion of Sebaey et al. [47], thicker specimens were considered by adding 0° plies in the cores of the laminates of each bending arm, to increase the bending stiffness. 

Eight new MD-DCB laminate configurations were defined, keeping the same combinations of interface angles but increasing the number of plies of the laminates. In all the cases the stacking sequences considered for the beams of the specimens are balanced and symmetrical. However, to compensate for differences in stiffness between the two arms of the specimen due to the different orientations of the interface plies (in addition to ensuring a symmetrical opening), six of these configurations have been defined with one arm thicker than the other. These additional plies have not been included for all the combinations of interface angles, but only for those with lower flexural–stiffness ratios. The eight new stiffer laminate configurations are presented in Table 3. As shown in the table, laminates L3_15_17, L4_15_18, L5_16_17, L7_19_21, L8_19_22, and L9_20_21 correspond to specimens with different beam thicknesses. In laminates L3 and L7, the total number of plies was the same as in laminates L2 and L6, 32 and 40 plies, respectively. In laminates L4, L5, L8 and L9, an additional 0° was added to one of the specimen beams.

**Figure 1:**
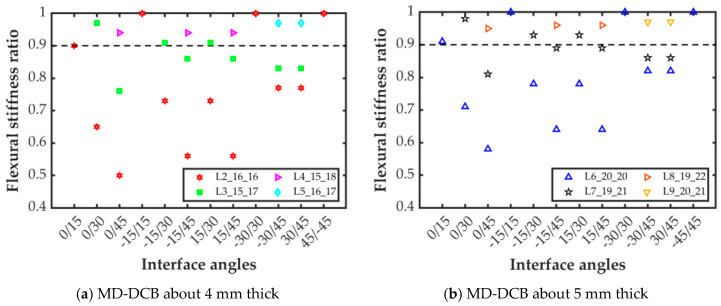
Flexural stiffness ratio for the interfaces and laminate configurations considered.

All laminate configurations, from the more flexible L2_16_16_θ_1__θ_2_ to the stiffer L9_20_21_θ_1__θ_2_, fulfilled the criterion established for the bending/twisting parameter, namely, *D*_c_ < 0.25. Moreover, the higher the bending flexural stiffness for each part of the specimen, the lower the value of the elastic parameter *B*_t_ (see Appendix A). Therefore, the assessment analysis focused on the ratio between the bending moduli of the specimen arms. Again, all laminate configurations with a flexural stiffness ratio equal to or higher than 0.9 were considered to be homogeneous flexural specimens. Flexural stiffness ratios below this value would result in specimens with unbalanced bending arms and asymmetric opening displacements, which would entail mixed-mode crack propagation. As shown in Figure 1a, eight interface combinations for laminate L2 did not fulfill this criterion (red hexagram below the dashed black line in the figure). Although the ratio for interface 0/15 is right in the limit, it is taken to be valid. Thus, these eight interface configurations are reevaluated by transferring one 0° ply from the stiffer specimen beam to the other one, which corresponds to laminate L3_15_17_θ_1__θ_2_. With this laminate configuration, three out of these eight interfaces fulfilled the flexural–stiffness ratio criterion (green squares above the dashed black line in the figure). For further improvement of the flexural stiffness ratio, an additional 0° layer was added to the specimen beam with the lowest flexural stiffness. So, these two new laminate configurations were taken into account: laminate L4_15_18_θ_1__θ_2_ for interfaces 0/45 and ±15/45, and laminate L5_16_17_θ_1__θ_2_ for interfaces ±30/45. As can be seen in Figure 1a, with the inclusion of these alternative laminate configurations, the value of the flexural–stiffness ratio was above the 0.9 limit for all the considered interfaces, and similar opening displacements can be expected in both specimen beams.

The same procedure as that of the specimens which were approximately 4 mm thick (around 32 plies) was likewise followed for the specimens that were approximately 5 mm thick (laminates L6 to L9). As shown in Figure 1, the flexural–stiffness ratio was higher for the thicker laminates (Figure 1b) than for the thinner ones (Figure 1a). However, the same eight interface configurations with a flexural–stiffness ratio lower than 0.9 for laminate L2 also do not fulfill the criterion for laminate L6. Again, transferring one of the plies from the stiffer beam to the more flexible one (laminate L7) served to achieve a flexural stiffness higher than 0.9 only for the same three interface configurations as for laminate L3. Adding an additional 0° layer to the more flexible arm of laminate L7, laminate L8, or laminate L9 ensured that all the interface combinations fulfilled the criterion on the flexural–stiffness ratio, as shown in the figure.

Table 4 summarizes the laminate configurations obtained for the different interface combinations considered during the numerical exploration campaign.

### 2.2. Finite Element Model

Before manufacturing and testing the MD-DCB specimens, different FE simulations were carried out to check if the interlaminar fracture toughness in multidirectional interfaces can be determined in the laminate and interface configurations selected in the previous section. The main objective of this numerical investigation was to ensure that the interlaminar crack propagates in the correct interface, and that there is no crack jumping or branching or any other form of damage.

**Figure 2:**
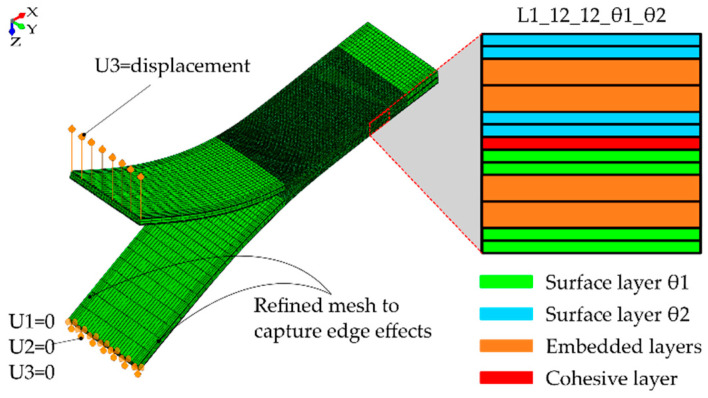
FE mesh and boundary conditions used to model the MD-DCB specimens with close-ups of through-the-thickness meshing.

The commercial FE software Abaqus/Standard 6.14 was used to model the proposed MD-DCB specimens based on the configuration indicated in the ISO-15024 [37] standard. The actual length and width of the specimens considered were 175 mm and 22.5 mm, respectively, and each had a precrack of 50 mm. However, to reduce the computational time, the specimen length was reduced to 100 mm, not including those parts of the specimen which were either away from the crack tip or not loaded during the test. This did not affect the local stresses close to the delamination area and, thus, did not alter the obtained results. The model was divided in three parts: the precrack zone (50 mm); an uncracked zone (40 mm), in which the propagation of the crack is simulated; and a final zone, where the two specimen beams are bonded with a tie constraint (10 mm). The mesh was refined both in the crack tip zone, to properly simulate the interlaminar damage propagation, and near the specimen edges, to correctly capture the edge effects (see Figure 2). The eight-node solid elements with incompatible modes C3D8I were used for all simulations. The beams of the specimens were modelled by defining one element of 0.125 mm in the thickness direction for each of the ±θ plies, and two central blocks to simulate the 0° layers, as shown in Figure 2.

Cohesive elements were used for simulating the propagation of the crack, including the cohesive element formulated by Turon et al. [52], and were then implemented by means of a user subroutine defined element (UEL). The cohesive elements had a negligible thickness of 1 μm. Both the cohesive element size and the entire delamination length were calculated according to what was indicated by Soto et al. [53]. Considering the lack of interlaminar fracture toughness properties for multidirectional interfaces with this material system, the values of *G*_Ic_ and *G*_IIc_ reported by Santos et al. [17] (i.e., 1.7 and 2.3 J/m^2^, respectively) were used for the modelling. The penalty stiffness was set as being equal to K_0_ = 1 × 10^6^ N/mm^3^, which should be large enough to avoid altering the compliance of the assembly. Finally, the interfacial strength was σ_0_ = 2.7 MPa. The ply elastic mechanical properties reported in Table 1 were also used for the FE model.

#### 2.2.1. LaRC04 Criterion for Matrix Cracking

For the investigation of crack jumping and crack branching during the propagation of the interlaminar crack, it was hypothesized that these two mechanisms were triggered using matrix cracking. Hence, the matrix tensile failure index was assessed using LaRC04 failure criteria [54] along the simulation. Whenever the matrix tensile failure index reaches one around the delamination plane, it is considered that crack jumping will occur during the experimental test. The LaRC04 in-plane tensile matrix cracking failure criterion was implemented by means of the ABAQUS user output variable standard subroutine, UVARM. The matrix tensile failure presented by Pinho et al. [54] corresponds to:

(2) $$FI=\left(1-g\right)\frac{\sigma_{22}}{Y_{is}^{T}}+g{\left(\frac{\sigma_{22}}{Y_{is}^{T}}\right)}^{2}+\frac{\frac{\tau_{12}^{2}}{G_{12}}+\frac{3}{2}\beta \tau_{12}^{4}}{\frac{S_{is}^{L^{2}}}{G_{12}}+\frac{3}{2}\beta S_{is}^{L^{4}}}$$

where $Y_{is}^{T}$ is the in situ transverse tensile strength, g is the material fracture toughness ratio $\left(\frac{{G_{I}}_{c}}{G_{II_{c}}}\right)$, $\sigma_{22}$ is the normal stress, $\tau_{12}$ is the shear stress, and β is a nonlinear relationship parameter between shear stress and shear strain, which was taken to equal 5 × 10^−8^, as it was by Sebaey et al. [47].

Notice that no damage evolution is defined and, thus, the specimen always remains elastic.

#### 2.2.2. Crack Front Assessment

During the propagation of an interlaminar crack, the shape of the crack front depends on the laminate stacking sequence and the interface angles. Multidirectional interfaces may result in a nonuniform distribution of the energy release rate and generate asymmetrical crack fronts [46], which can also facilitate the migration of the crack to another interface planes. On the other hand, the crack propagation was experimentally measured on the specimen edge and the crack front was considered as a straight line along the specimen width. Such an assumption may not be reliable, especially for multidirectional composites with asymmetrical crack fronts. Hence, the elastic bending–twisting and bending–bending couplings may cause highly curved thumbnail-shaped delamination fronts.

**Figure 3:**
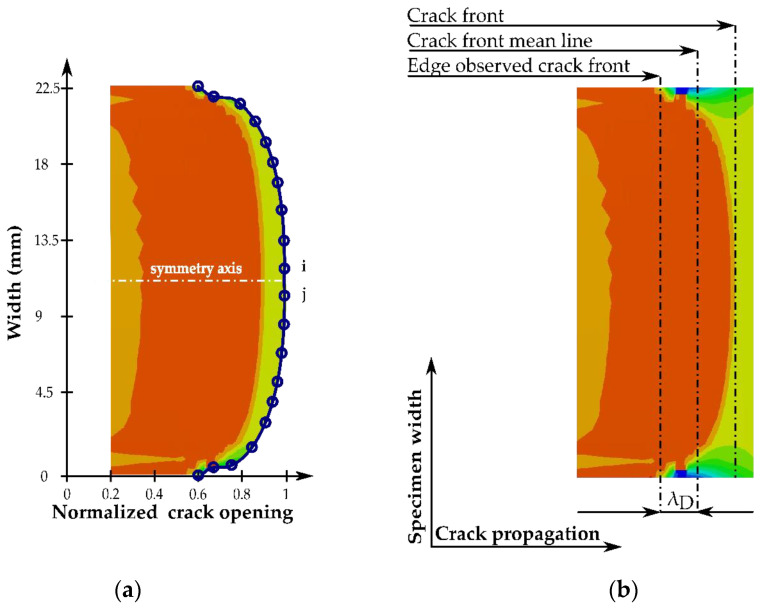
Schematic representation of a predicted crack front and crack opening during the simulations for the determination of: (**a**) the Out-Of-Symmetry parameter, and (**b**) the visual deviation parameter.

In this study, the symmetry of the crack front was assessed by obtaining the out-of-plane stress distribution right ahead of the crack tip during the simulations, as can be schematically seen in Figure 3. Then, the Out-Of-Symmetry (OOS) parameter defined by Sebaey et al. [47] was determined according to the following procedure: (i) the crack opening (just behind the crack front, where the maximum out-of-plane stress is experienced) was measured and normalized with respect to its maximum value; (ii) a symmetry line was drawn at the specimens’ midwidth (see Figure 3a); and (iii) the absolute value of the difference in the crack opening between each pair of equidistant nodes (node *i* and *j*) was labeled as the out of symmetry (OOS) coefficient. This procedure was repeated for all pair of nodes in the crack front, and the maximum value was reported as the OOS coefficient of the specimen.

The straightness of the crack front was quantified by defining the visual deviation $\lambda_{D}$ parameter as the difference between the relative crack position at the specimen edge and at the crack front mean line, as can be seen in see Figure 3b. The $\lambda_{D}$ parameter was used to establish a quantitative comparison and discussion of the relative position of the crack front between the proposed specimen configurations.

### 2.3. Numerical Simulation Results

**Figure 4:**
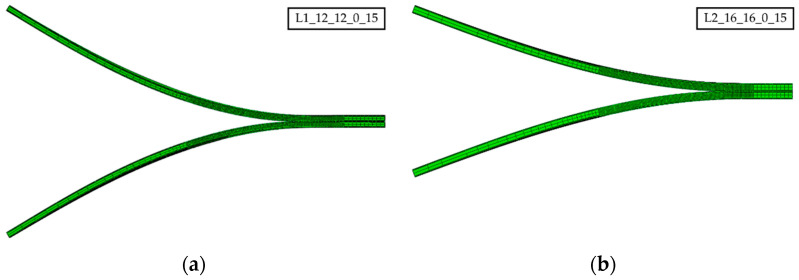

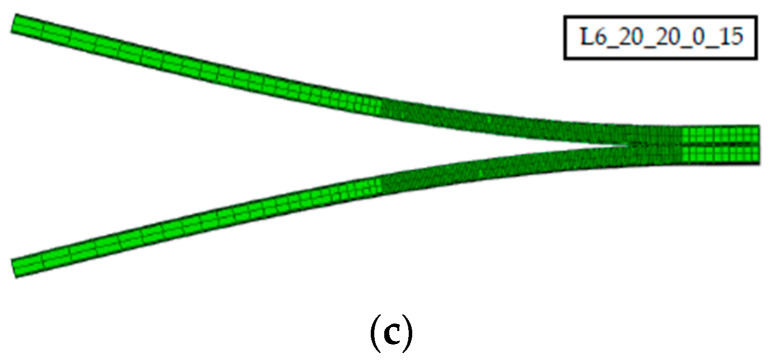
Symmetric opening for the MD-DCB specimens at the maximum crack propagation for laminates (**a**) L1_12_12_0_15, (**b**) L2_16_16_0_15, and (**c**) L6_20_20_0_15.

The computational time for each model was on average about 18 and 27 h, employing a cluster of 12 and 6 CPUs, respectively. Prior to the exploration of the failure index for all proposed specimen configurations (Table 4), the deformed shapes of the specimen arms were analyzed to warranty symmetric opening and pure mode I crack propagation. All laminate configurations, from the most flexible (L1) to the stiffest (L8 and L9) specimens, showed appropriate crack opening. This can be observed in Figure 4 for the configurations with the lowest flexural–stiffness ratios, namely L1_12_12*_*0_15, L2_16_16*_*0_15, and L6_20_20*_*0_15, at the maximum crack propagation (see Appendix A).

#### 2.3.1. Evaluation of Matrix Cracking

**Figure 5:**
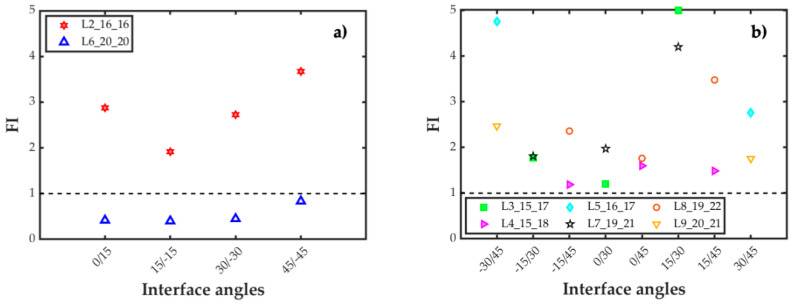
Failure Index (FI) for the MD-DCB specimens, with respect to the interface angles and stacking sequence: (**a**) beam arms with same thickness, and (**b**) beam arms with different thicknesses.

Since the value of the matrix tensile Failure Index (FI) obtained for the three interface configurations considered for laminate L1 (Table 4) was higher than one, the numerical results for laminate L1 have not been reported, for the sake of conciseness. The matrix cracking FI for all interface angles and laminate combinations modelled (from L2 to L9) are summarized in Figure 5. The results for the MD-DCB specimens with balanced number of plies in the bending arms, L2 and L6, are shown in Figure 5a, where a clear numerical pattern can be identified: the higher the bending stiffness, the lower the FI. The value of FI was higher than one for all the considered interface combinations in the case of laminate L2, with an average FI equal to 2.8. For laminate L6, all interface combinations achieved a FI value below 1, and the average was 0.5. This means that, for the considered interface combinations, neither matrix cracking nor crack jumping should be expected for laminate L6. By contrast, crack jumping cannot be ruled out for laminate L2 and the considered interface angles.

Figure 5b shows the FI results for laminates with arms with different thicknesses. It can be observed that the value of FI is higher than one for all cases; thus, matrix cracking and crack jumping can be expected for all of them. Moreover, no clear trend can be observed in function of the bending stiffness. While FI decreased with bending stiffness for laminates L5 and L9, the opposite tendency was observed for laminates L4 and L8, and no tendency was observed for laminates L3 and L7.

After the analysis of the failure index for all the considered laminate–interface combinations, it can be concluded that for the MD-DCB specimens with a balanced number of plies, the failure index can be reduced by increasing the stiffness of the arms. The same trend was found by Sebaey et al. [47] for a different material and interface combination. However, for the specimens with an unbalanced number, there was no such a tendency, and it was not possible to determine an effective way to reduce the failure index. Consequently, only laminate L6 and interfaces 0/15, 15/−15, 30/−30 and 45/−45 have been taken for the rest of work, as these were the only combinations that achieved a failure index below 1.

#### 2.3.2. Crack Front Symmetry and Visual Deviation

Table 5 summarizes the values of OOS, $\lambda_{D}$, and FI that were obtained with the simulations of the interface combinations considered for laminate L6. The parameter $\lambda_{D}$ was in the range between 0.91 and 0.95 mm, which was relatively constant for all cases. Hence, a common average value can be adopted, to be added to the measured crack length during the propagation test to correct the crack front shape. On the other hand, the values of OOS varied between 2.2 and 12.5%. As can be seen in the table, the minimum values of $\lambda_{D}$ and OOS were found in the 45/−45 interface, while the maximum values of the two parameters were found in the 15/−15 interface. The inverse situation was found for the failure index, with the minimum for the 15/−15 interface and the maximum for the 45/−45 one. Thus, there was no direct relation between either the symmetry or straightness of the crack front and the value of the failure index for matrix cracking.

To guarantee a successful subsequent experimental campaign, a safety factor equal to two was introduced for evaluating the matrix tensile FI of L6 laminate. Consequently, the MD-DCB specimens should be manufactured using laminate L6, and interfaces 0/15, 15/−15 and 30/−30.

It is worth remarking that for all laminate and interface cases, the value of FI quickly increased from zero to the final values reported in Figure 5 and Table 5 within a relatively short crack extension. Hence, the authors considered that, during the experimental campaign, the specimens should be tested directly, without extending the precrack a few millimeters before the test, as indicated in the standards. This is to guarantee enough experimental crack extension and data for characterizing the initiation and propagation fracture toughness value without any crack jumping or any other form of damage.

## 3. Manufacturing and Testing of the MD-DCB Specimens

This section describes the manufacturing process of the MD-DCB specimens using the Markforged MarkTwo^®^ 3D printer, as well as the experimental test campaign to obtain the mode I fracture toughness in the 0/15, 15/−15 and 30/−30 interfaces.

As mentioned before, the materials used in this work were the 3D-printed composite materials obtained with the Markforged MarkTwo^®^ filament, combining a thousand continuous carbon fibers with PolyAmide 6, CF/PA. The fiber volume fraction reported in the literature varies between 27 and 41%, with matrix and fiber dominated areas and high void content [14,15,55,56]. Summaries of both the in-plane elastic properties and the interlaminar fracture toughness values for unidirectional specimens are presented in Table 1.

The MarkTwo^®^ 3D printer requires that the bottom and top most plies and the outer walls of the printed parts must be printed with either pure PA filament or Markforged^®^ PA filament that has been reinforced with short carbon fiber (CF 12% wt. [57]), known as Onyx^®^. In this case, the second option was used. The number of the outer Onyx® walls was set to one, while the system imposes four floor layers (the same number is required of the top layers, but the printing process was stopped before, so as to avoid it). However, to avoid any influence in the results, the Onyx^®^ floor plies and walls were removed from the specimens before the tests. Owing to the high amount of carbon fiber reinforcement, and in order to avoid bonding problems between specimen and building platform during the printing process, all specimens were manufactured individually with round corners and an extended Onyx^®^ brim. The nominal thickness of the CF/PA and Onyx^®^ plies was 0.125 mm.

**Figure 6:**
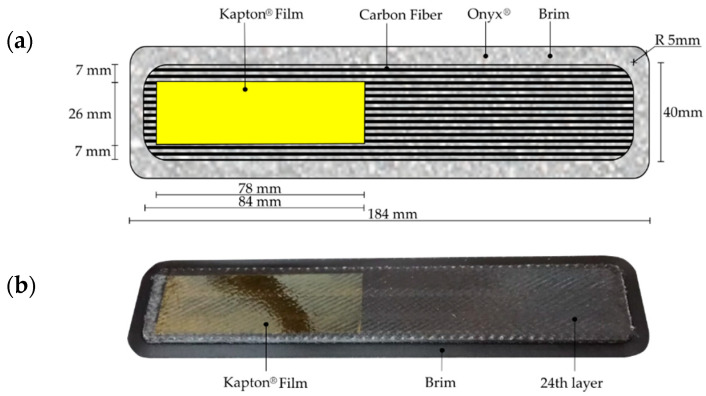
Manufacturing of the MD-DCB specimens with: (**a**) schematic top view of a specimen with the corresponding printed part dimensions, and (**b**) placement of the adhesive Kapton^®^ tape at midplane, after the 24th layer.

The MD-DCB specimens for the experimental exploration of the interlaminar fracture toughness in mode I were designed and printed according to the ISO-15024 [37] standard. According to the results of the numerical simulations (Section 2.3), the specimens should have two 2.5 mm thick beams (5 mm in total) following the stacking sequence of laminate L6. To generate the precrack at the specimen’s midplane, once the bottom half was fully printed (after the 24th deposited layer), the printing process was paused. Then, an adhesive Kapton^®^ tape was laid on top of the bottom half of the specimen with the adhesive surface in contact with the bottom half-printed part to generate the starter crack, see Figure 6a. Special attention was paid to avoid any wrinkle in the Kapton^®^ tape that could generate fiber obstruction in the nozzle during the deposition process. An additional area without film beyond the precrack area and around the lateral edges was left, in order to ensure good adhesion of the subsequent deposited layers (see Figure 6b). Notice that the additional area without film had to be removed/cut before testing, as it would prevent the propagation of the crack.

After manufacturing, the brim was manually removed, and all the printed specimens were stored inside a dry box with several desiccant bags at room temperature. This was to prevent moisture absorption in the nylon, and avoid any possible influence on the experimental results, such as a reduction in strength and stiffness with the increment in ductility and impact resistance observed in moisturized specimens [58,59,60]. Six MD-DCB specimens for each of the three interface combinations based on laminate L6 selected in Section 2.3.2 were manufactured, prepared, and tested.

Before the tests, the edges of the specimens were cut with a diamond saw, removing the material in excess of what was needed for the good adhesion of the 25th layer, i.e., the layer on top of the Kapton^®^ tape. Similarly, the additional 6 mm of material in front of the Kapton^®^ tape was also cut. As commented before, the printing process was stopped after the last CF/PA layer was deposited in the sample (44th ply); this was so due to the risk that the Onyx^®^ plies might deform in a different way and debond from the CF/PA plies, which would invalidate the test, as was reported by Santos et al. [17]. Therefore, the four bottom Onyx^®^ plies were cut with the same diamond saw before the test. The final dimensions of the interlaminar specimens after the post-processing were 175 mm × 25 mm × 5 mm (length, width, and thickness) and 50 mm (precrack).

**Figure 7:**
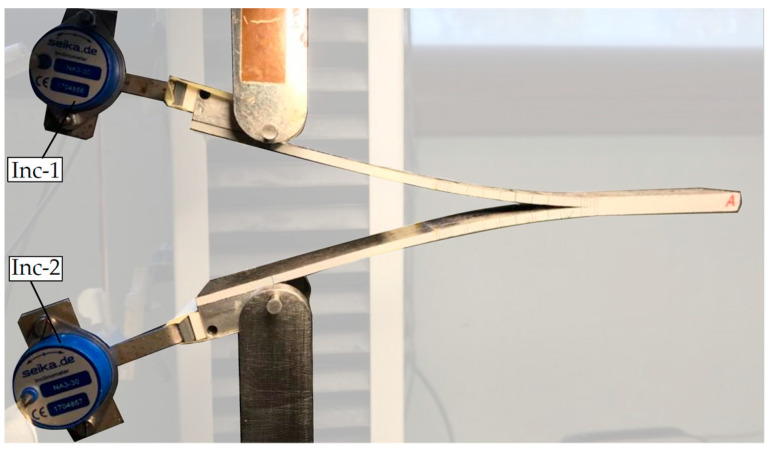
MD-DCB specimen during testing instrumented with inclinometers (Inc-#).

To facilitate tracking the crack propagation during the test, the edges of the specimens were painted with an appropriate white paint, and the propagation lines indicated in the standard were marked. The area destined for the loading blocks in the top and bottom surfaces of the MD-DCB samples was abraded using an 80-grade sandpaper creating a longitudinal and transversal texture. Aluminum MW2001-6 loading blocks were adhered to the specimens by coating the surface with Henkel Loctite^®^ EA 9466™ epoxy adhesive (USA). The adhesive was left to cure at room temperature for 24 h using hand-presses to fix sample and loading blocks properly. To determine the interlaminar fracture toughness in mode I by means of the J-integral method (Paris [61] and Sarrado et al. [62]), one NA3-30 inclinometer (with a resolution of 0.005°) was attached to each loading block to monitor their inclination during the test. The instrumented post-processing specimen is shown in Figure 7.

All fracture tests were carried out using an MTS (Eden Prairie, Minnesota, MN, USA) Insight testing machine, equipped with a 5 kN load cell at 23 ± 2 °C and 50 ± 5 HR. All tests were displacement controlled with a crosshead speed of 1.5 mm/min during loading, and 25 mm/min for unloading. The loading speed was modified with respect to the one considered in the ISO-15024 [37] standards, i.e., 5 mm/min, as a more appropriate response was found during these preliminary tests.

It is worth mentioning that, for the entire experimental campaign, the precrack was not extended from the insert, and all tests began from the Kapton^®^ adhesive tape. This was because the numerical predictions of the matrix tensile FI quickly increased within a short crack propagation length. Therefore, performing precrack operation during the experimental campaign might increase the possibility of not obtaining enough valid experimental data for calculating the *G*_Ic_. To account for this, in this study, the initiation fracture toughness (*G*_Ic,ini_) was measured after the crack had been extended by 5 mm, while the propagation one (*G*_Ic,prop_) was calculated from this point until the last crack length without experiencing unstable crack propagation and/or crack jumping.

## 4. Experimental Results and Discussion

### 4.1. Experimental Load-Displacement Curves

**Figure 8:**
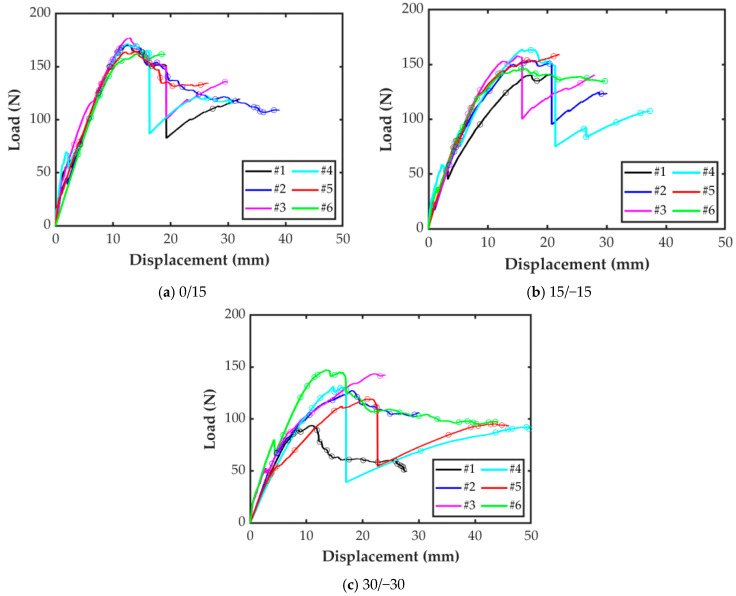
Load-displacement curves for the MD-DCB specimens. Crack propagation points are indicated on the curves.

The load-displacement curves for the tested specimens are shown in Figure 8. It is worth mentioning that for the sake of clarity, the curves show the entire loading (including load drops) until the instant prior catastrophic unstable crack growth occurs.

As can be seen in Figure 8a, all the 0/15 specimens showed a similar initial behavior, and the first crack propagation took place at a bending force around 70 N for all of them. However, some of these specimens (0/15-1, 0/15-2, and 0/15-4) experienced an average load drop of 9 N in the elastic region. This batch of specimens had an averaged strength of 172 N, after this, load-displacement curve exposed a nonlinear softening response. Thus, all the specimens can be used to determine the value of the mode I interlaminar fracture toughness of this interface combination.

The load-displacement curves of 15/−15 specimens, reported in Figure 8b, showed less repetitiveness than in the case of the 0/15 specimens, as well as a lower average strength of 148 N. A load drop is also observed for all the specimens, except for the 15/−15-5 one. The response for specimen 15/−15-1 varied after this initial load drop, resulting in a behavior that was less stiff than the rest of the batch. Thus, this specimen will not be taken into account for the determination of *G*_Ic_ in the 15/−15 interface.

Figure 8c shows the load-displacement curves for the 30/−30 specimens. A large variability in the behavior of the specimens can be observed. On the one hand, specimens 30/−30-1 and 30/−30-2 experienced a clean and smooth crack propagation along the entire test with similar stiffness in the linear elastic region. However, the nonlinear response within the failure process zone was different for the two specimens, and the peak load for the latter was about 50% higher. The response for specimen 30/−30-3 was similar to that of specimen 30/−30-2, but the peak load was even higher. An unstable crack growth took place right after this point. On the other hand, specimens 30/−30-4 and 30/−30-5 showed a similar stiffness at the beginning, but after a load drop at 5 mm of opening the stiffness of the specimen 30/−30-5 was significantly lower than that of the rest. Finally, specimen 30/−30-6 showed a stiffer response if compared to the other samples, especially from the 5 mm of opening displacement. Thus, specimens 30/−30-1, 30/−30-4, and 30/−30-5 will not be taken into account to determine the value of the mode I interlaminar fracture toughness of the 30/−30 interface combination.

### 4.2. Analysis of the Crack Propagation Modes

**Figure 9:**
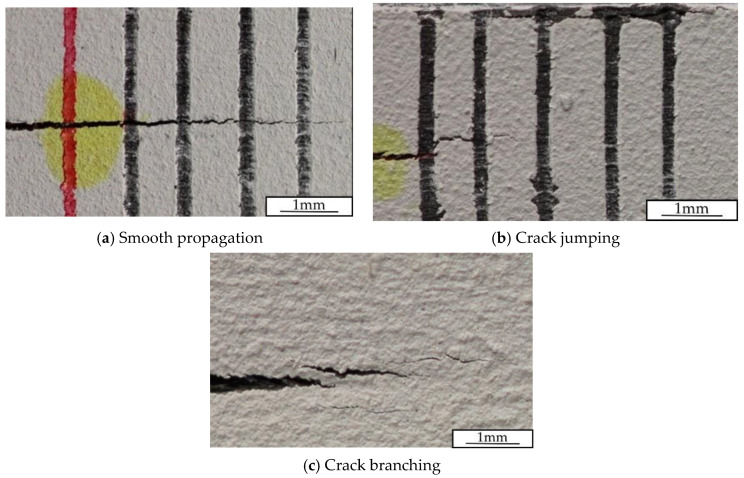
Different delamination propagation modes observed during the MD-DCB tests.

**Figure 10:**
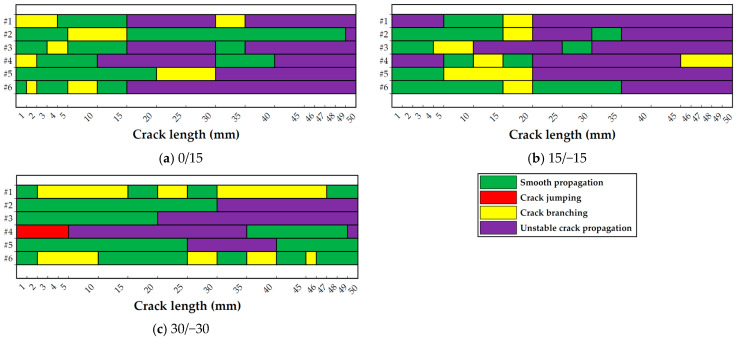
Crack propagation modes identified in each specimen during the MD-DCB tests. The symbol “#” indicates the specimen number for each interface configuration.

During the characterization of the interlaminar fracture toughness using the MD-DCB specimens, four crack propagation modes were identified: smooth propagation, unstable crack propagation, crack branching, and crack jumping propagation. Figure 9 shows graphical examples of these modes, except for unstable crack propagation. During the tests, all specimens experienced more than one crack propagation failure mode, as has been schematically represented in Figure 10 for the six specimens tested per interface combination.

As can be observed in Figure 10, all 0/15 and 15/−15 specimens experienced smooth crack propagation, crack branching, and unstable crack propagation at some moment, though to different extents. Specimens 0/15-2, 0/15-3, 0/15-5, and 0/15-6 showed smooth propagation for some millimeters before crack branching appeared (see Figure 10a). Except for specimen 0/15-5, smooth crack propagation reappeared after some extension under crack branching. The initial crack propagation mode for specimens 0/15-1 and 0/15-4 was crack branching; however, after some extension under this mode, smooth crack propagation appeared. Unstable crack growth occurred in all the 0/15 specimens between 10 and 30 mm of crack length, except for specimen 0/15-2. However, for specimens 0/15-1, 0/15-3, and 0/15-4, this propagation mode alternated between smooth crack propagation and crack branching. In the case of specimen 0/15-2, unstable crack growth appeared after 49 mm of extension, right at the very end of the test.

In the case of the 15/−15 specimens, the initial crack propagation mode found was smooth propagation, except for specimens 15/−15-1 and 15/−15-4. For these two last specimens, unstable crack propagation was initially experienced, followed by smooth crack propagation (see Figure 10b). In all cases, crack branching appeared after some extension under smooth crack propagation, followed by unstable crack growth, with the exception of specimens 15/−15-4 and 15/−15-6, where a smooth crack propagation reappeared before unstable growth. Smooth crack propagation also reappeared for specimens two and three, with crack lengths ranging from 30 to 35 mm and 25 to 30 mm, respectively. Specimen 15/−15-4 experienced crack branching for the last 5 mm of the test, instead of unstable crack propagation. Thus, all specimens achieved at least 15 mm crack length before unstable crack propagation, except for specimen 0/15-4, which only had 10 mm of stable crack growth.

As can be seen in Figure 10c, the crack extension in specimens 30/−30-1 and 30/−30-6 alternated between smooth crack growth and crack branching, but no unstable crack propagation appeared. In the case of specimens 30/−30-2, 30/−30-3, and 30/−30-5, unstable crack propagation appeared after several millimeters of extension under smooth crack growth. For the latter, smooth crack propagation reappeared for the last 10 mm of crack extension. In the case of specimen 30/−30-4, the initial crack extension implied crack jumping and the crack ultimately migrated to another plane, invalidating the rest of the test.

Although crack propagation modes other than smooth crack propagation were observed for all the specimens, crack jumping (or plane migration) was found only in one case. This corroborates the validity of the process we designed for the specimens to specifically avoid crack jumping.

### 4.3. Mode I Interlaminar Fracture Toughness

After analyzing the resulting load-displacement curves and crack propagation modes for all the specimens, it can be concluded that not all of them should be considered for determining the interlaminar fracture toughness in the considered interface angles. Essentially, all specimens with a clearly distinct behavior from the rest of the batch were not considered for the analysis, as indicated previously. In addition, it is worth remarking that the zones with unstable crack growth or crack jumping have not been considered for the determination of the interlaminar fracture toughness.

In the case of the 0/15 interface, all specimens up to the unstable crack growth can be considered. For the 15/−15 interface, specimens with initial unstable crack growth (i.e., 15/−15-1 and 15/15-4) are not considered for the determination of the interlaminar fracture toughness. Specimens 15/−15-2, 15/−15-5, and 15/15-6 experienced at least 20 mm of stable crack propagation and similar response, so they will be included in the analysis. Although for specimen 15/−15-3, the crack only propagated a few millimeters under smooth conditions, it has been also considered. Finally, for the 30/−30 interface, taking into account the disparity of the resulting load-displacement curves and the resulting crack propagation modes for these specimens, only specimens 30/−30-2, 30/−30-3, and 30/−30-6 have been considered. Although specimen 30/−30-1 presented several millimeters of stable crack growth, the corresponding peak load was very low compared to the rest. Similarly, the stiffness response of specimen 30/−30-5 differed from the other specimens. Finally, specimen 30/−30-4 experienced crack jumping from the beginning of the test.

Table 6 summarizes the initiation and propagation of interlaminar fracture toughness in mode I, calculated using the J-integral method for the three MD-DCB interface angles analyzed in this work. As no extension of the precrack was performed before the tests, the initiation value *G*_Ic,ini_ was designated as being 5 mm of crack extension. The propagation fracture toughness, *G*_Ic,prop_, was calculated from the initiation point until the last crack length of stable growth.

**Figure 11:**
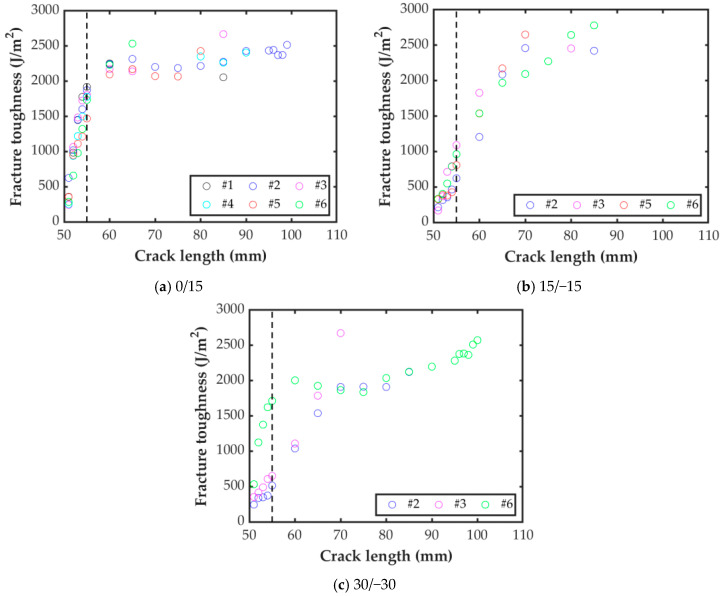
Crack propagation resistance curves for the MD-DCB specimens.

The propagation resistance curves (R-curves) for the 0/15, 15/−15, and 30/−30 MD-DCB specimens are presented in Figure 11. The markers in the curves correspond to the crack propagation lengths stated in the standard [37]. The vertical black dashed line at 55 mm indicates the point considered for crack onset. For the 0/15 interface (Figure 11a), the fracture toughness initially increased with the crack length, until a plateau is reached (between 60 and 80 mm of crack extension), which was in good agreement with the average propagation value reported in Table 6 (2146 J/m^2^). For crack lengths longer than 80 mm, a slight increase was found.

The R-curve for the four representative specimens of the 15/−15 interface is shown in Figure 11b. The four curves show that the fracture toughness increased with the crack length for short cracks. Unlike the 0/15 case, there was no clear plateau in the central range of values, but *G*_Ic_ tended to stabilize around 2550 J/m^2^. In any case, the average fracture toughness propagation value reported in Table 6 (1720 J/m^2^) can be considered as a conservative value for this case.

As expected, the scatter for the R-curve for the 30/−30 interface was higher than for the other two interface combinations (see Figure 11c). From the beginning up to 15 mm of crack length, specimens 30/−30-2 and 30/−30-3 followed a similar trend, while the initial values of *G*_Ic_ for specimen 30/−30-6 were much higher. However, after a crack growth of 15 mm, the specimens with a similar trend were 30/−30-2 and 30/−30-6, with a plateau up to a crack length of about 85 mm. Meanwhile, the interlaminar fracture toughness for specimen 30/−30/-3 was much higher for its last measured point before unstable propagation. Similar to what has been observed for the 0/15 case, after the plateau, the value of the mode I interlaminar fracture toughness increased with crack length, although in this case this was only for one specimen, 30/−30-6.

The current study presents several limitations, as is explained next. The delamination test avoided the crack jumping during the entire opening, triggering a desirable response as stated in the standard, validating the characterization. However, all tested specimens experienced different crack propagation failure modes during testing, such as crack branching, unstable crack propagation, and smooth propagation. Thus, the obtained toughness values should be carefully considered for designing components with this material. Moreover, the experimental cases presented have been limited to 0/15, 15/−15, and 30/−30 interface orientations. More interface combinations should be explored, including angle orientations larger than 45°, to further characterize the fracture response of the CF/PA printed composite material.

### 4.4. Fractographic Analysis

A fractographic analysis of the representative MD-DCB postmortem specimens was carried out after the tests. All specimens were manually slit open, and the first 20 mm crack propagation length was cut without damaging the original failure morphology with the use of the same diamond saw (Section 3). A S4100 Scanning Electron Microscope (SEM) (Hitachi Co., Ltd., Tokyo, Japan) was used to analyze the microscopic failure characteristics, with an accelerating voltage of 7 kV. Prior to the observation, each specimen’s bottom surface was affixed with conductive tape, and its conductivity was improved by evaporating carbon through K950 (Emitech SA., Germany) for better scanning quality.

**Figure 12:**
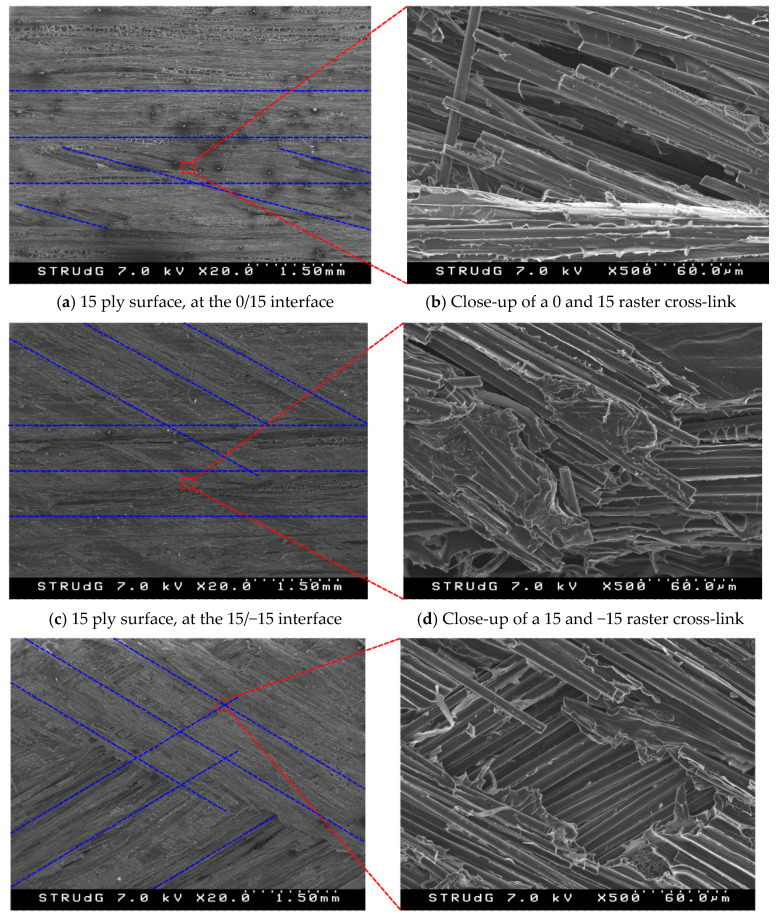
SEM images of the fracture surface of the representative specimens. Dashed blue lines indicate the filament raster directions.

The fractographic analysis of the 15° ply at the 0/15 interface (Figure 12a) revealed furrows between filament rasters, in addition to areas with air bubbles or voids. Some loose long broken fibers could also be observed on the surface, as well as white PA particles surrounded by small dark areas. As can be observed, the fiber rasters were not completely straight, but instead had a longitudinal waviness shape. Rasters at 0 and 15° were present on the surface (marked with dashed blue lines), although the first are placed at a higher level (brighter). This might indicate that the crack did not grow completely flat at the 0/15 interface, but rather in a combination of 0 and 15° cross-linking rasters. One of the more evident instances of interface cross-linking has been analyzed under a higher magnification in Figure 12b. These 15° oriented fibers have been pulled-out from the resin and broken during delamination, while the resin around the longitudinal fibers experienced more plastic deformation.

The fracture surface of 15° ply at the 15/−15 interface showed fewer voids and broken fibers (Figure 12c) than those observed in the 0/15 interface. Again, furrows were observed along of adjacent filament rasters, which had a slight waviness of shape in both continuous interfacing layers (mark with dashed blue line). Again, cross-linking of rasters in the two directions could be observed, although the ones in the −15° direction seemed to be close to the 0° direction. Few traces of pulled-out fibers were observed, most of them in the 15° rasters. Analyzing one of the cross-linking zones at a higher magnification in Figure 12d, a group of fragmented 15° fibers can be observed after being pulled-out, with plastic deformation in the surrounding matrix.

The fracture surface of the −30° layer at interface 30/−30 is shown in Figure 12e. Matrix dominated areas and voids can be observed, as well as many broken fibers. As for the other two interfaces, cross-linking of the two raster directions can be clearly observed, indicating that the propagation plane along the width of the specimen was not completely flat. Figure 12f shows the amplification of one cross-link between the 30° and the −30° rasters, where it can be observed that a central area of the 30° fibers has been removed from the surface. This micrography also showed that the little matrix observed around the pulled-out fibers had experienced plastic deformation. Amplifying this intersection, fibers located at a higher level in the image showed traces of the debonding fibers on the PA and fewer broken fibers; a polygonal area of this high ply was removed during test. The lower fibers were almost dry, with irregular space between them. PA plastic deformation is observed in form of ridge.

The fractographic analysis revealed that all specimens shared common characteristics, such as matrix and fiber dominated areas, voids along adjacent filament fiber rasters, and waviness in the fiber rasters. The cross-linking of rasters in different directions was also observed for the three analyzed interface combinations. This was probably due to the fact the delamination did not propagate in a flat plane across the width of the specimen, but rather with waving between the rasters of the adjacent interface plies.

**Figure 13:**
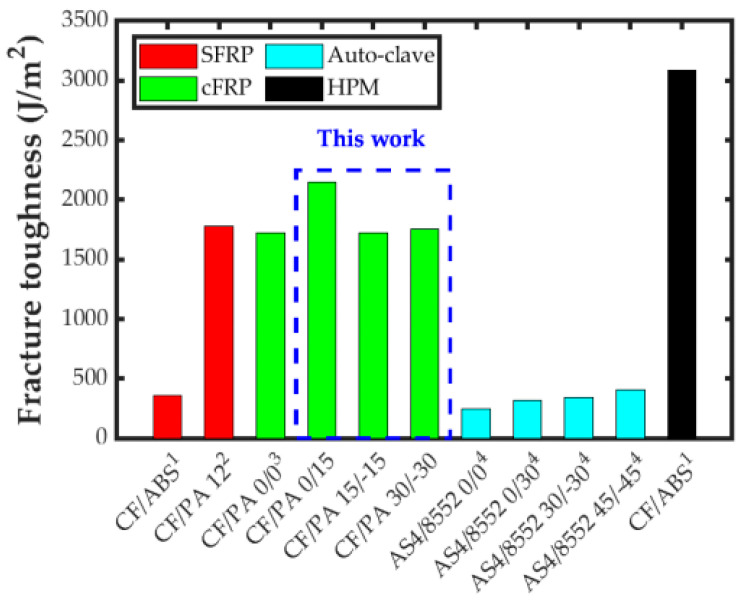
Graphical qualitative comparison of the interlaminar fracture toughness of different materials and manufacturing technologies using DCB test: ^1,2^ SFRP composites with CF 15% wt [30,31]; ^3^ unidirectional and MD cFRP composites of CF/PA [17]; ^4^ Fiber Reinforced Polymer (FRP) AS4/8552 [63]; and ^1^ Hot-Press Molded (HPM) using 3D printing materials [30].

Finally, to put the results of this study into perspective, the obtained values of fracture toughness in this work have been compared with the reported ones for thermoplastic composites using other manufacturing technologies and materials. Figure 13 shows a qualitative comparison of the propagation mode I interlaminar fracture toughness of SFRP, cFRP, and Hot-Press Molded (HPM) using 3D printed composite systems, as well as traditional prepreg composites cured in autoclave [17,30,31,63]. On the one hand, the SFRP and HPM composites were made of either CF 15% wt reinforcing ABS or PolyAmide 12 (PA 12), respectively. On the other hand, the prepreg material was the unidirectional carbon/epoxy (Hexcel AS4/8552) with a fiber volume fraction of 57% [64]. It is worth remarking that this is a qualitative comparison since different thermoplastic and thermoset matrices were included, as well as continuous and discontinuous reinforcements.

In the figure, it can be clearly observed that the mode I interlaminar fracture toughness was lower for the composites with thermoset matrix cured in autoclave than for most of the ones with thermoplastic matrix. Only in the case of ABS reinforced with short CF was the interlaminar fracture toughness comparable to that of the thermoset ones. Despite thos, a clear tendency can be observed for the traditional laminated composite: the higher the mismatch angle at the interface, the higher the fracture toughness. The value of the fracture toughness for the PA matrix reinforced with short CF was similar to that of the cFRP with 0/0 interface obtained in this study. In fact, this was also true for the cFRP composites with 15/−15 and 30/−30 interfaces. On the other hand, the cFRP composite with the 0/15 interface in this study showed the highest toughness value of all AM cases. Finally, it is worth remarking that using the same short CF/ABS material, the value of the mode I interlaminar fracture toughness increased by more than six times when a manufacturing process was used where a material compaction would be applied, such as for the HPM process. Actually, this was the composite that obtained the highest toughness. Therefore, the compaction stage can considerably improve the mechanical performance of parts, since the flaws created during the entire deposition process can be reduced.

## 5. Conclusions

An experimental campaign was carried out using the FE method to evaluate the probability of crack jumping during the MD-DCB test. The simulations employed a cohesive zone model to predict the onset and propagation of the delamination for nine laminates, with symmetrical and balanced bending arms. The LaRC04 matrix tensile failure criterion was employed to establish if crack jumping was triggered or not during the test. This numerical exploration predicted four interfaces with a failure index lower than one, according to one single laminate configuration. After imposing an additional safety factor of two, three different interfacing angles were experimentally characterized, so as to validate the numerical methodology proposed in this study. The delamination test showed four crack propagation modes, which led us to determine the interlaminar fracture toughness for all cases. To better understand the fracture mechanics, the SEM microscopic morphology of the delaminated surfaces were analyzed. Hence, the following conclusions can be drawn from the investigation:

- MD-DCB specimens with similar flexural stiffness in both arms (to guarantee pure mode I opening) were used to characterize the interlaminar fracture toughness in multidirectional interfaces, avoiding crack jumping. Only one of the 18 tested specimens showed this type of crack propagation, and only for 5 mm.
- The mismatch angle at the interface played a key role for the fracture characterization; the lower this mismatch angle, the higher the level of repetitiveness. Hence, the 0/15 interface showed a clear trend for load-displacement and R-curves. The tendency was not so clear for the 15/−15 interface, and even less so for the 30/−30 one.
- The value of the measured mode I interlaminar fracture toughness depended on the mismatch angle of the interface. For the 15/−15 and 30/−30 cases, the propagation values of *G*_Ic_ (1720 and 1758 J/m^2^, respectively) were virtually the same to that measured by Santos et al. [17] for the same material (1720 J/m^2^) with unidirectional interfaces. However, the measured value for the 0/15 interface was higher, at 2146 J/m^2^.
- The fractographic analysis of all the interfaces revealed that the crack propagated not on a flat plane across the width of the specimen, but rather with waving between the rasters of the adjacent interface plies. Pulled-out and broken fibers for all raster directions could be detected for the three interface combinations. In addition, resin rich areas and areas with high density of fibers were observed in all cases, along with voids, especially for the 0/15 case.

Future work will expand this study by characterizing the interlaminar fracture toughness of the same printed CF/PA composite under mode II and mixed mode loading.

## Figures and Tables

**Table 1:** Material properties and standard deviation of the additively continuous carbon-fiber-reinforced thermoplastic composite [17]. (* These properties were reported by Iragi et al. [16]).

Property	
Longitudinal elastic modulus, *E*_11_ (GPa)	66.5 ± 7.1
Transversal elastic modulus, *E*_22_ (GPa)	6.2 ± 1.1
In-plane Poisson ratio, $\nu_{12}$	0.39 ± 0.03
In-plane shear modulus, *G*_12_ (GPa)	2.1 ± 0.2
Fracture toughness mode I, *G*_Ic_ (J/m^2^)	1.7 ± 0.1
Fracture toughness mode II, *G*_IIc_ (J/m^2^)	2.3 ± 0.1
Longitudinal tensile strength, *X*^T^ (MPa)	752 ± 88.6
Longitudinal compressive strength, *X*^C^ (MPa)	426 ± 9.7 *
Transverse tensile strength, *Y*^T^ (MPa)	49.3 ± 9.9
Transverse compressive strength, *Y*^C^ (MPa)	66 ± 6.6 *
Shear strength, *S*^L^ (MPa)	31 ± 0.1
Ply thickness, *t* (mm)	0.125

**Table 2:** Assessment of the bending/twisting coupling effects for both the two arms and the entire specimen of laminate L1_12_12_θ_1__θ_2_. Symbols **✓** and **✗** indicate if the condition is fulfilled or not (*D*_c_ < 0.25). B and T stand for bottom and top arms, respectively, while A stands for the whole laminate.

θ_1_/θ_2_	*D*_c_			θ_1_/θ_2_	*D*_c_		
	B	T	A		B	T	A
0/15	✓	✓	✓	15/30	✓	✓	✓
0/30	✓	✓	✓	15/45	✓	**✗**	✓
0/45	✓	**✗**	✓	−30/30	✓	✓	✓
−15/15	✓	✓	✓	−30/45	✓	**✗**	✓
−15/30	✓	✓	✓	30/45	✓	**✗**	✓
−15/45	✓	**✗**	✓	−45/45	**✗**	**✗**	✓

**Table 3:** Redefined stacking sequences for the MD-DCB specimens. θ_1_ and θ_2_ correspond to the pair combination between 0, ±15, ±30, and ±45 interfacing angles. Symbol // indicates the initial delamination plane, and $ indicates odd symmetry.

Codification	Stacking Sequence
L2_16_16_θ_1__θ_2_	[(±θ_1_/0_6_)_s_//(±θ_2_/0_6_)_s_]
L3_15_17_θ_1__θ_2_	[(±θ_1_/0_6_)_$_//(±θ_2_/0_7_)_$_]
L4_15_18_θ_1__θ_2_	[(±θ_1_/0_6_)_$_//(±θ_2_/0_7_)_s_]
L5_16_17_θ_1__θ_2_	[(±θ_1_/0_6_)_s_//(±θ_2_/0_7_)_$_]
L6_20_20_θ_1__θ_2_	[(±θ_1_/0_8_)_s_//(±θ_2_/0_8_)_s_]
L7_19_21_θ_1__θ_2_	[(±θ_1_/0_8_)_$_//(±θ_2_/0_9_)_$_]
L8_19_22_θ_1__θ_2_	[(±θ_1_/0_8_)_$_//(±θ_2_/0_9_)_s_]
L9_20_21_θ_1__θ_2_	[(±θ_1_/0_8_)_s_//(±θ_2_/0_9_)_$_]

**Table 4:** Laminate configurations and interface combinations considered for the numerical simulation campaign.

Codification	Interface Angles *(*θ_1_/θ_2_*)*
L1_12_12_θ_1__θ_2_	0/15, −15/15, −30/30
L2_16_16_θ_1__θ_2_	0/15, −15/15, −30/30, −45/45
L3_15_17_θ_1__θ_2_	0/30, −15/30, 15/30
L4_15_18_θ_1__θ_2_	0/45, −15/45, 15/45
L5_16_17_θ_1__θ_2_	−30/45, 30/45
L6_20_20_θ_1__θ_2_	0/15, −15/15, −30/30, −45/45
L7_19_21_θ_1__θ_2_	0/30, −15/30, 15/30
L8_19_22_θ_1__θ_2_	0/45, −15/45, 15/45
L9_20_21_θ_1__θ_2_	−30/45, 30/45

**Table 5:** The out of symmetry (OOS) coefficient, visual deviation ($\lambda_{D}$), and matrix tensile Failure Index (FI) for the MD-DCB specimens of laminate L6.

Laminate	OOS (%)	*λ*_D_ (mm)	FI
L6_20_20_0_15	8.3	0.92	0.42
L6_20_20_15_−15	12.5	0.95	0.40
L6_20_20_30_−30	8.3	0.92	0.45
L6_20_20_45_−45	2.2	0.91	0.91

**Table 6:** Initiation and propagation interlaminar fracture toughness in mode I for the MD-DCB specimens.

Laminate	Number of Specimens	Fracture Toughness (J/m^2^)	
		Onset	Propagation
L6_20_20_0_15	6	1763 ± 159	2146 ± 100
L6_20_20_15_−15	4	870 ± 71	1720 ± 251
L6_20_20_30_−30	3	960 ± 655	1758 ± 345

## Data Availability

The raw/processed data required to reproduce these findings can be obtained from the corresponding author upon request.

## Appendix A. Results of the Flexural Analysis

The following tables summarize the results of the flexural analysis for all the interface combinations for each laminate configuration. The tables include the results for the evaluation of the *B*_t_ and *D*_c_ elastic parameters for the three sections of the specimens, as well as the bending stiffness for each bending arm of the specimen, and the corresponding flexural–stiffness ratio.

**Table A1:** Results of the flexural analysis for the laminate configuration L1_12_12_θ_1__θ_2_.

θ_1_/θ_2_	*B*_t_			*D*_c_			*E*_f_ (GPa)		
	B	T	A	B	T	A	B	T	Ratio
0/15	0	0.031	0.003	0.01	0.06	0.02	6.6	5.7	0.86
0/30	0	0.054	0.005	0.01	0.23	0.05	6.6	3.7	0.56
0/45	0	0.057	0.004	0.01	0.27	0.06	6.6	2.6	0.39
−15/15	0.031	0.031	0	0.06	0.06	0.04	5.7	5.7	1
−15/30	0.031	0.054	0	0.06	0.23	0.07	5.7	3.7	0.65
−15/45	0.031	0.057	0	0.06	0.27	0.07	5.7	2.6	0.45
15/30	0.031	0.054	0.009	0.06	0.23	0.07	5.7	3.7	0.65
15/45	0.031	0.057	0.008	0.06	0.27	0.07	5.7	2.6	0.45
−30/30	0.054	0.054	0	0.23	0.23	0.11	3.7	3.7	1
−30/45	0.054	0.057	0.001	0.23	0.27	0.12	3.7	2.6	0.69
30/45	0.054	0.057	0.011	0.23	0.27	0.12	3.7	2.6	0.69
−45/45	0.057	0.057	0	0.27	0.27	0.12	2.6	2.6	1

**Table A2:** Results of the flexural analysis for the laminate configuration L2_16_16_θ_1__θ_2_.

θ_1_/θ_2_	*B*_t_			*D*_c_			*E*_f_ (GPa)		
	B	T	A	B	T	A	B	T	Ratio
0/15	0	0.018	0.002	0.01	0.05	0.02	15.8	14.1	0.9
0/30	0	0.03	0.003	0.01	0.17	0.04	15.8	10.3	0.65
0/45	0	0.029	0.002	0.01	0.19	0.04	15.8	7.94	0.5
−15/15	0.018	0.018	0	0.05	0.05	0.03	14.1	14.1	1
−15/30	0.018	0.03	0	0.05	0.17	0.05	14.1	10.3	0.73
−15/45	0.018	0.029	0	0.05	0.19	0.06	14.1	7.94	0.56
15/30	0.018	0.030	0.005	0.05	0.17	0.05	14.1	10.3	0.73
15/45	0.018	0.029	0.005	0.05	0.19	0.06	14.1	7.94	0.56
−30/30	0.03	0.03	0	0.17	0.17	0.08	10.3	10.3	1
−30/45	0.03	0.029	0	0.17	0.19	0.08	10.3	7.94	0.77
30/45	0.03	0.029	0.006	0.17	0.19	0.08	10.3	7.94	0.77
−45/45	0.029	0.029	0	0.19	0.19	0.09	7.94	7.94	1

**Table A3:** Results of the flexural analysis for the laminate configuration L3_15_17_θ_1__θ_2_.

θ_1_/θ_2_	*B*_t_			*D*_c_			*E*_f_ (GPa)		
	B	T	A	B	T	A	B	T	Ratio
0/30	0	0.026	0.003	0.01	0.16	0.04	13	12.6	0.97
0/45	0	0.025	0.002	0.01	0.18	0.04	13	9.95	0.76
−15/30	0.020	0.026	0	0.05	0.16	0.05	11.5	12.6	0.91
−15/45	0.020	0.025	0	0.05	0.18	0.06	11.5	9.95	0.86
15/30	0.020	0.026	0.005	0.05	0.16	0.05	11.5	12.6	0.91
15/45	0.020	0.025	0.005	0.05	0.18	0.06	11.5	9.95	0.86
−30/45	0.034	0.029	0	0.18	0.19	0.09	8.2	9.9	0.83
30/45	0.034	0.025	0.006	0.18	0.18	0.09	8.2	9.9	0.83

**Table A4:** Results of the flexural analysis for the laminate configuration L4_15_18_θ_1__θ_2_.

θ_1_/θ_2_	*B*_t_			*D*_c_			*E*_f_ (GPa)		
	B	T	A	B	T	A	B	T	Ratio
0/45	0	0.022	0.002	0.01	0.17	0.04	13	12.2	0.94
−15/45	0.02	0.022	0	0.05	0.17	0.06	11.6	12.2	0.94
15/45	0.02	0.022	0.004	0.05	0.17	0.06	11.6	12.2	0.94

**Table A5:** Results of the flexural analysis for the laminate configuration L5_16_17_θ_1__θ_2_.

θ_1_/θ_2_	*B*_t_			*D*_c_			*E*_f_ (GPa)		
	B	T	A	B	T	A	B	T	Ratio
−30/45	0.030	0.025	0	0.17	0.18	0.08	10.2	9.95	0.97
30/45	0.030	0.025	0	0.17	0.18	0.08	10.2	9.95	0.97

**Table A6:** Results of the flexural analysis for the laminate configuration L6_20_20_θ_1__θ_2_.

θ_1_/θ_2_	*B*_t_			*D*_c_			*E*_f_ (GPa)		
	B	T	A	B	T	A	B	T	Ratio
0/15	0	0.011	0	0.01	0.04	0.02	30.9	28.2	0.91
0/30	0	0.019	0.002	0.01	0.13	0.03	30.9	21.9	0.71
0/45	0	0.017	0.001	0.01	0.15	0.04	30.9	17.9	0.58
−15/15	0.011	0.011	0	0.04	0.04	0.03	28.2	28.2	1
−15/30	0.011	0.019	0	0.04	0.13	0.04	28.2	21.9	0.78
−15/45	0.011	0.017	0	0.04	0.15	0.05	28.2	17.9	0.64
15/30	0.011	0.019	0.003	0.04	0.13	0.04	28.2	21.9	0.78
15/45	0.011	0.017	0.003	0.04	0.15	0.05	28.2	17.9	0.64
−30/30	0.019	0.019	0	0.13	0.13	0.06	21.9	21.9	1
−30/45	0.019	0.017	0	0.13	0.15	0.07	21.9	17.9	0.82
30/45	0.019	0.017	0.004	0.13	0.15	0.07	21.9	17.9	0.82
−45/45	0.017	0.017	0	0.15	0.15	0.07	17.9	17.9	1

**Table A7:** Results of the flexural analysis for the laminate configuration L7_19_21_θ_1__θ_2_.

θ_1_/θ_2_	*B*_t_			*D*_c_			*E*_f_ (GPa)		
	B	T	A	B	T	A	B	T	Ratio
0/30	0	0.017	0.002	0.01	0.13	0.03	26.4	25.8	0.98
0/45	0	0.015	0.001	0.01	0.14	0.04	26.4	21.3	0.81
−15/30	0.013	0.017	0.003	0.04	0.13	0.04	24.1	25.8	0.93
−15/45	0.013	0.015	0	0.04	0.14	0.05	24.1	21.3	0.89
15/30	0.013	0.017	0.003	0.04	0.13	0.04	24.1	25.8	0.93
15/45	0.013	0.015	0.003	0.04	0.14	0.05	24.1	21.3	0.89
−30/45	0.021	0.015	0	0.14	0.14	0.07	18.4	21.3	0.86
30/45	0.021	0.014	0.003	0.14	0.13	0.07	18.4	21.3	0.86

**Table A8:** Results of the flexural analysis for the laminate configuration L8_19_22_θ_1__θ_2_.

θ_1_/θ_2_	*B*_t_			*D*_c_			*E*_f_ (GPa)		
	B	T	A	B	T	A	B	T	Ratio
0/45	0	0.014	0	0.01	0.13	0.04	26.4	25.2	0.95
−15/45	0.013	0.014	0.003	0.04	0.13	0.05	24.1	25.2	0.96
15/45	0.013	0.014	0.003	0.04	0.13	0.05	24.1	25.2	0.96

**Table A9:** Results of the flexural analysis for the laminate configuration L9_20_21_θ_1__θ_2_.

θ_1_/θ_2_	*B*_t_			*D*_c_			*E*_f_ (GPa)		
	B	T	A	B	T	A	B	T	Ratio
−30/45	0.019	0.015	0.003	0.13	0.14	0.07	21.9	21.3	0.97
30/45	0.019	0.015	0.003	0.13	0.14	0.07	21.9	21.3	0.97
